# A 26-Year Experience in Chorionic Villus Sampling Prenatal Genetic Diagnosis

**DOI:** 10.3390/jcm3030838

**Published:** 2014-07-24

**Authors:** Paula Jorge, Maria Manuela Mota-Freitas, Rosário Santos, Maria Luz Silva, Gabriela Soares, Ana Maria Fortuna

**Affiliations:** 1 Center of Medical Genetics Doutor Jacinto Magalhães, Oporto Hospital Center, C.H.P., EPE, Praça Pedro Nunes, 88, 4099-028 Porto, Portugal; E-Mails: manuela.freitas@chporto.min-saude.pt (M.M.M.-F.); rosario.santos@chporto.min-saude.pt (R.S.); m.luz.silva@chporto.min-saude.pt (M.L.S.); gabriela.soares@chporto.min-saude.pt (G.S.); ana.fortuna@chporto.min-saude.pt (A.M.F.); 2 Unit for Multidisciplinary Research in Biomedicine, UMIB, ICBAS-UP, Rua Jorge Viterbo Ferreira 228, 4050-313 Porto, Portugal

**Keywords:** chorionic villus sampling, prenatal diagnosis, prenatal referrals, maternal age, fetal loss, chromosomal abnormality/aneuploidy, maternal cell contamination, monogenic disorders

## Abstract

This report describes the trends of chorionic villus sampling (CVS) referred for prenatal genetic diagnosis in the past two and a half decades in a Portuguese Center. Our cohort of 491 CVS was mostly performed by the transcervical method at the 12th gestational week. Data collected within the framework of this study relate to the following: sampling method, referral reason *versus* abnormality and incidence of procedure-related pregnancy loss, that declined to about 0.5% over the last 15 years. The year 2000 represented a change in referral reasons for chorionic tissue collection, shifting from almost exclusively for cytogenetic testing to an increasing number of molecular tests for monogenic disorders. Herein, success rates as well as cytogenetic and/or molecular DNA results are presented. These latter include not only tests for several monogenic disorders, but also aneuploidy and maternal cell contamination screening. This retrospective analysis reiterates that CVS is a safe and reliable first trimester technique for prenatal diagnosis in high genetic risk pregnancies.

## 1. Introduction

In the early 1980s, unlike the prevailing tendency in other European countries, prenatal testing had not been implemented in Portugal. Although the Institute of Medical Genetics (now named Center of Medical Genetics, CGM) had the knowhow and the essential equipment to carry out the laboratory tests, the Director of the Institute (Doctor Jacinto de Magalhães: 1938–1987), envisaged prenatal diagnosis (PND) integrated in a multidisciplinary facility and proposed the establishment of a public facility with services of genetic counseling, collection of fetal products, genetic and biochemical routine tests and medical termination of pregnancy (the latter carried out in a collaborating central Hospital). At that time, however, prenatal practices where considered ethically inappropriate; abortion due to genetic causes was not permitted nor was there any legislation concerning this matter. Following years of legal, ethical and political disputes, it was in May 1984 that the Portuguese government approved a law that “excludes the illicitness of the voluntary termination of pregnancy for genetic causes”, enabling the beginning of PND at the CGM. This was a landmark for the progress in the fetal diagnosis of genetic disorders in Portugal. Our Center was initially a nationwide service provider, but later become essentially regional as further PND facilities were set up in other parts of the country [1,2].

The continuous clinical implementation of CVS started in 1983, developed by Denis Fairweather and Humphrey Ward, of the University College of London, for the diagnosis of the hemoglobinopathies [3,4]. CVS worldwide popularity and widest application was attained when chromosomal analysis by the direct preparation of tissue was reported, beginning a new era of fetal genetic diagnosis [5]. Two different approaches were available for chorionic tissue sampling: the transcervical and the transabdominal methods. Each method has its own followers but both require trained, coordinated and skilled human resources [6,7]. In either case, procedures are performed under ultrasound guidance, without any type of anaesthesia and in an ambulatory setting. Irrespective of the sampling method, the fetal damage and/or loss directly related to this invasive procedure were the most serious complications of CVS [8,9]. It is now consensual that the number of procedure-induced limb defects is insignificant if sampling occurs after the 10th week of gestation [10,11]. When opting for an invasive procedure, other complications to take into account are twin pregnancies and mosaicism confined to the placenta [12,13]. This sampling procedure is performed four to six weeks earlier in gestation, when compared to amniocentesis. The relative anticipation of results reduced couple anxiety and allowed access to pregnancy termination at a safer gestational time (in some countries pregnancy termination for genetic causes was, in the first years, allowed only until the 16th week). CVS was introduced at CGM in 1988, on a nationwide basis, performed by trained obstetricians and rapidly became a “must” for pregnant women, particularly those with advanced maternal age (AMA) expecting a first child [1,2].

In a CVS a small sample of chorionic (placental) tissue is obtained; however, the cells need to be further prepared before analysis. Three methods can be used in cytogenetic laboratories: the direct preparation of uncultured cytotrophoblast cells, the short and the long-term culture methods. The latter is less prone to false-positive and false-negative results, in addition to providing better quality metaphase preparations and is thus preferred for chromosome analysis. Due to the risk of confined placental mosaicism, false-positive results can be observed, which has the disadvantage of causing anxiety until the final result is obtained [12,14,15].

The ease of obtaining DNA from non-cultured villi guided the development of polymerase chain reaction (PCR)-based molecular techniques (QF-PCR, quantitative fluorescent PCR) for rapid identification of the most frequent aneuploidies [16,17,18]. Around 2000, in our Center, a multiplex-PCR was implemented, using a set of 20 microsatellite markers (short tandem repeats, STR), for the screening of common aneuploidies, detection of maternal cell contamination (MCC), fetal sexing and zygosity determination, allowing an earlier result in ongoing pregnancies [19].

## 2. Materials and Methods

This is a retrospective and descriptive study of 491 CVS specimens received in our Center, between January 1988 and December 2013, collected in-house or sent from other PND Centers. For the present report the authors used private records and the Prenatal Diagnosis Center Annual Activity Reports (1987–2007) as well as data in clinical files provided by geneticists/physicians following pregnant women in external clinical units (1987–2013). The data was either obtained from publicly available records or anonymized before disclosure to researchers, waiving the need for ethic board approval [1,2]. Sampling in our Center was performed mostly by the transcervical method (75%) and a satisfactory amount of material was obtained on first attempt in 83.8% of cases. The procedure was carried out at a mean of 11 weeks plus 3 days (mode = 12 weeks), the earliest case being collected at 6 weeks plus 2 days and the latest two at the 17th week of gestation. The age of the pregnant women had a mean of 34 years and 4 months, ranging from 17 to 49 years of age. Consanguinity was reported in three couples (0.61%).

Referrals in the cohort were for risk of a monogenic disorder, advanced maternal age (AMA, defined as age 35 years or older), ultrasound abnormality, family history of chromosome abnormality, couple anxiety, ultrasound marker and positive biochemical maternal serum screening. In fifty-eight cases there was a combination of two reasons for referral. Furthermore, DNA studies were performed in clinically selected cases of extreme maternal anxiety, high aneuploidy risk due to a specific ultrasound marker or risk for an X-linked disorder. After 2004, all samples with a normal female karyotype were analyzed for MCC using STR markers located on chromosomes 13, 18, 21 and X. 

### Chromosome Preparations and DNA Extraction

For both cytogenetic and molecular studies the villi were separated from maternal tissue under an inverted microscope and cleaned in an appropriate wash medium. After separation, chromosome preparations were obtained by a direct and/or a long-term culture method following previously described procedures with slight modifications [20,21]. Metaphases were initially banded using trypsin-Giemsa technique, but this was more recently substituted by the Leishman stain. DNA extraction and additional studies were done according to standard procedures and disease-specific techniques [22]. A blood drop from all pregnant women was also collected onto filter paper, for MCC testing and/or further DNA analyses, if required.

## 3. Results and Discussion

The birth rate in Portugal has decreased drastically over this 26-year period; between 1988 and 2013 there has been a 30.5% decrease in live-births [23]. In this period a total of 21,304 prenatal invasive procedures were processed in our Center, of which 491 (2.3%) were CVS. A comparison of the annual CVS and amniocenteses shows a fall in the decade between 1994 and 2003, to less than 1% CVS per total invasive procedures. This fact is attributed to the introduction of amniofiltration (early amniocentesis) and the first trimester ultrasound examination (11–13 weeks), with the offer of amniocentesis in case of a positive screen. In contrast, in the last three years CVS represented approximately 20% of all invasive procedures (30% in 2012) [1,2].

The major drawback of CVS is procedure-related pregnancy loss, with published estimates varying between 0.7% and 2% [24]. Even though a quality ultrasound had enabled CVS to attain a high-level of efficacy and safety, with the advantage that it can be carried out earlier than amniocentesis (9–13th gestational week), procedure-related complications are emphasized to the parents. The rate of CVS-related pregnancy loss, defined as fetal loss occurring within 15 days after the procedure, in the first sixteen years exceeded by 2.6% that obtained in the last decade. In fact, in the latter period our CVS-related pregnancy loss rate has reached values similar to those of amniocentesis (0.5%), although simple comparison of the rate of fetal loss after CVS *versus* amniocentesis does not seem a suitable method for determining the procedure-related risk of loss, because CVS is performed earlier in pregnancy. A possible contributing factor to the decrease in post-procedure losses is the lower number of attempts needed to obtain a satisfactory amount of villi, which in turn is correlated with operators’ experience and skills. Again, in the last decade, there was a 12% decrease in the number of cases in which more than one attempt was required. There was no recorded case of fetal limb reduction/defects in our cohort. When an inconclusive result was obtained women were offered an amniocentesis. In cases referred for a monogenic disorder the protocol included karyotyping.

Overall, the referrals for CVS were comprised of 49.5% for cytogenetic analysis, 50.3% for monogenic disorders and 0.2% due to maternal anxiety (non-medical). In our cohort there were 31 sampling/culture failures (6.3%). Cytogenetic and/or molecular results of the remaining 460 cases are presented herein. MCC was observed in 8 of the analyzed samples (1.7%). A summary of the 109 (23.7%) affected cases and respective maternal age is presented in Table 1.

**Table 1:** Number of affected cases among the 460 chorionic villus sampling (CVS).

*N* = 109	*N* (%)	Mean Maternal Age ± SD (Range)
Chromosome abnormality	28 (6.1)	32.0 ± 6.6 (18–45)
Monogenic disease	81 (17.6)	31.2 ± 4.8 (18–40)

**Figure 1:**
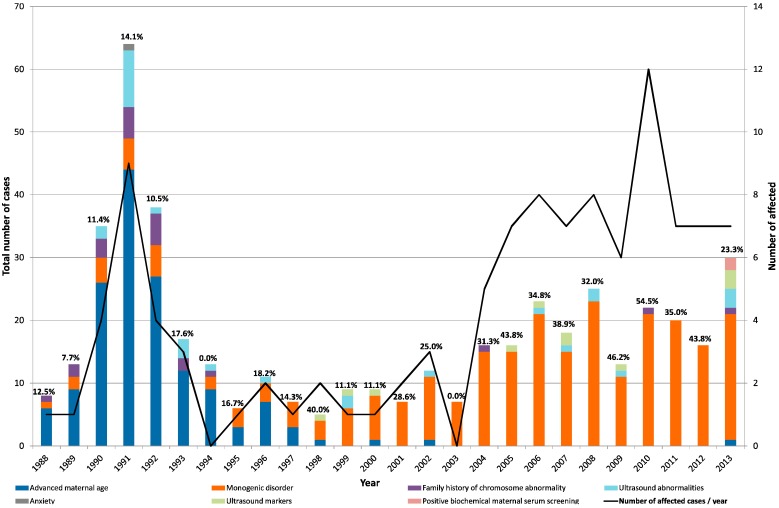
Annual variability of referral reasons among the 460 CVS. Number of referrals is represented in the *Y*-axis distributed by years, while the second *Y*-axis represents number of cases where a diagnosis was attained (affected). Percentage of affected cases per year is shown on graphic.

Data on the annual distribution of referral reasons as well as number and proportion of affected cases are shown in Figure 1. In cases with more than one referral reason, only that with the higher clinical impact was graphically represented.

In the period under study, average maternal age of our population, at pregnancy, increased from 27 to 32 years [23]. AMA has currently been dropped as indication for invasive PND; nevertheless, maternal age was our main referral until 1997 and according to published studies it is the only etiological factor that correlates with frequency of aneuploidies [25]. Among the 28 chromosomal abnormalities and the 81 monogenic disorders detected, comparison of maternal age at sampling revealed that similar results were observed in the two groups and both are below what was considered advanced maternal age (cut-off 35 years), indicating an a priori low risk population (Table 1). Referral bias might contribute to this apparent discrepancy, given the fact that our institution specializes in genetic diseases and in the beginning of the analyzed time period it was the only health service in the country that offered CVS. A large proportion of pregnant women were referred to our Center because of a high risk for a genetic disorder, whether previously known from family history or detected *de novo* in the ongoing pregnancy. Especially in more recent years, with increasing availability of Prenatal Centers, women with low risk factors (AMA alone) were not usually referred to us. Because genetic risks for familial chromosomal anomalies and monogenic disorders are not associated with maternal age, this fact probably shifts the median age of women to younger values. Other related factors are the small number of samples analyzed and the fact that most women referred for AMA alone, mostly had amniocentesis and not CVS.

The year 2000 represented a shift in referral reasons for CVS collection, moving from almost exclusively for cytogenetic testing to an increasing number of molecular tests for monogenic disorders. This was mainly attributed to the development and rapid spread of DNA-based methodology. In fact, in the decade of 2004–2013 the positivity rate reached on average 40% with a maximum of 55% in 2010, largely due to genetic diagnosis of monogenic diseases.

Specific chromosomal analysis referrals consisted of AMA (*n* = 150), ultrasound abnormality (*n* = 28), family history of chromosome abnormality (*n* = 22), ultrasound marker (*n* = 11), positive biochemical maternal serum screening (*n* = 2) and anxiety (*n* = 1). Normal chromosome results were obtained in 187 of 214 cases (87.4%) at risk for a chromosomal aberration. Table 2 summarizes the karyotyping results stratified by age and referral reason.

Type and rate of numerical or structural chromosomal abnormalities found were similar to that of several previous studies [25,26]. Numerical anomalies were found in twenty cases and included ten trisomy 21, four trisomy 18, four monosomies X, one trisomy 20 and one triploidy. Among the patients with a fetus affected with trisomy 21 there were three recurrent situations and two of them were from the same woman. Trisomy 20 was observed in one direct culture and was proved to be a placenta-confined full trisomy, where a follow-up amniocentesis revealed a normal male karyotype with a normal outcome at delivery. Structural rearrangements were observed in eight cases: five inherited balanced translocations—t(5;14)mat, t(7;8)mat, t(7;14)pat, rob(15;22)pat, and rob(13;14)mat—the latter with a previous fetus with trisomy 13; two derivative chromosomes from parental balanced translocations—der(7)t(7;18)pat and der(18)t(18;20)mat—and one ring chromosome—r(21). Among the balanced structural anomalies there was a peculiar case of 45,XX,rob(15;22)pat, confirmed on follow-up amniocentesis, found in a fetus at risk for familial amyloidotic polyneuropathy (affected mother). Two cases of placenta-confined mosaicism were identified in subjects referred for monogenic disorders.

**Table 2:** Karyotyping results stratified by age and referral reason.

*N* = 215 * in 460	*N*	Age < 35	Age ≥ 35	Mean ± SD (Min–Max)	Referral Reason	*n*
**Normal**	187 ^#^	61	120	38.5 ± 5.5 (19–49)	AMA	145
					Ultrasound abnormality	19
					Family history of chromosome abnormality	13
					Ultrasound marker	7
					Positive biochemical maternal serum screening	2
					Anxiety	1
**Abnormal**	28	16	12	32.0 ± 6.6 (18–45)		
Trisomy 21	10	4	6	34.6 ± 6.3 (23–42)	Ultrasound marker	3
					Previous child with Down syndrome	3
					Ultrasound abnormality	2
					AMA	2
Trisomy 18	4	2	2	33.0 ± 9.3 (23–45)	Ultrasound abnormality	2
					AMA	1
					Ultrasound marker	1
Trisomy 20	1	0	1	41	AMA	1
Monosomy X	4	4	0	29.2 ± 6.5 (18–34)	Ultrasound abnormality	4
Triploidy	1	0	1	37	Ultrasound abnormality	1
Balanced structural rearrangement	5	4	1	30.9 ± 3.4 (25–35)	Parent carrier of balanced translocation	4
					* FAP	1
Unbalanced structural rearrangement	3	2	1	31.3 ± 4.0 (28–37)	Parent carrier of balanced translocation	2
					AMA	1

^#^ Age unknown in *n* = 6; * One case that was not referred for chromosomal abnormality.

Examples of the most frequent disorders screened through molecular studies include familial amyloidotic polyneuropathy (FAP, *n* = 39), Spinal Muscular Atrophy (SMA, *n* = 18), Fragile-X syndrome (FXS, *n* = 15) and Duchenne muscular dystrophy (DMD, *n* = 10). This is not surprising given that these are relatively frequent genetic disorders in most populations. There is an endemic cluster of FAP in the north of Portugal and SMA carrier frequency is also relatively high in our population [27,28,29,30]. One couple was referred in two consecutive pregnancies for SMA and Huntington’s disease (HD); the first fetus was affected with HD and the second, 46,XY was normal for both disorders.

Although dependent on amount of starting material, DNA yield in chorionic villi is higher than that obtained from uncultured amniocytes, thereby allowing reliable DNA analysis within hours/days of sampling, with the concomitant advantages. With the exception of FXS, nearly all molecularly diagnosed disorders were performed on uncultured villi. In eight out of fifteen cases of PND of FXS, long-term cultures were needed for methylation-sensitive Southern blot analysis, which is critical for distinguishing between a full mutation (expansion with >200 CGGs) responsible for the syndrome itself, and a large premutation (expansion with 56–200 CGGs) responsible for other Fraxopathies. In most cases, the sizing of the expansion was sufficient to determine the genotype. However, in two cases a subsequent amniocentesis was needed for analysis of the methylation status of the *FMR1* promoter due to a limitation inherent to sample type and timing of sampling: methylation of the full mutation is not always completed at the 8–10th week of gestation [31]. These two cases were proven to be *FMR1* size-mosaics with a premutation and a full mutation.

When CVS specimens are used directly there is an increased risk of MCC. Although samples undergo microscope-guided separation of the maternal deciduas, it is often difficult to eliminate all maternal tissue. For this reason, it is important to exclude MCC and be aware that the cells analyzed can be potentially different from those analyzed in cultured villi. In fact, in one particular case where the tissue was divided for FAP diagnosis and for cytogenetic analysis, both subcultures used for chromosome analysis consisted exclusively of maternal cells, while in the fragment used for FAP diagnosis MCC was completely excluded. This rare result represents a pitfall, highlighting the need to perform MCC exclusion in all aliquots, regardless of the studies they are submitted to.

Data on MCC is difficult to compare with other studies not only due to limited number of cases but also because reports of MCC seem to be based solely on cytogenetic analyses, where numbers are reported only when an XY constitution is observed. Because MCC cannot be cytogenetically identified in most pregnancies of female fetuses, since 2004 we implemented an STR-based molecular analysis for MCC exclusion in cases where disease-specific techniques were unable to detect contamination. Besides MCC screening, these analyses were also performed in other specific cases: ultrasound observation of a marker for aneuploidy involving chromosomes 13, 18, 21 or X, maternal anxiety or at risk pregnancy for an X-linked disorder (for fetal sexing until 2006). Out of the 176 CVS specimens received after 2004, sixty-eight cases were analyzed by a multiplex-PCR using a group of 20 STR markers, located on chromosomes 13, 18, 21 and X [19]. Results of the aneuploidy molecular screening were in accordance with those obtained upon karyotyping (16 normal and 4 affected) and in agreement with the expected relative incidence (two cases of monosomy X and two with trisomy 21).

In a pregnant carrier of a mutation in the *MTM1* gene (responsible for X-linked myotubular myopathy) fetal sexing was initially done, as a female result would avoid the need for further studies. This case, however, was found to be a male fetus hemizygous for the familial *MTM1* mutation. This case dates back to 2006; more recently, the possibility to perform fetal sexing by non-invasive prenatal testing (NIPT) avoids the need for fetal sampling [32].

The present study has limitations given that it is retrospective and reports a single-center experience, with biased and limited numbers, including follow-up after delivery, thereby hindering extrapolation to the national health public system. Nonetheless, it has the advantage of resulting from a large number of pregnancies from the same Center, with well-known policy changes over time.

## 4. Conclusions

This retrospective analysis of 491 cases reiterates that CVS has become a safe and reliable choice for first trimester prenatal diagnosis in pregnancies at risk for chromosomal abnormality and for genetic disorders (with the exception of a few tests such as FXS full mutation detection). These conclusions are based on the marked decrease of CVS-related fetal loss and number of attempts needed for successful sampling, particularly is the last decade. Regarding the laboratory handling of the sample, in our experience, it is important that all aliquots of the villi be tested for MCC before further studies.

Although there has been a dramatic decrease in pregnancies/live-births accompanied by an increase in maternal age at first conception, this is not reflected in the results reported herein. Over the last decade in our Center there was an increase in CVS number and in the proportion of affected cases among low-risk chromosomopathy pregnancies (including AMA), mostly due to referral bias. The increase in sampling as well as in positivity rate reflects an improvement in referral procedures which culminate in a more targeted PND intervention.

## Acknowledgments

The authors would like to thank Rui Vaz Osório (the Director of Institute of Medical Genetics between 1987 and 2001) for providing private records and Maria José Ortigão (Department of Informatics at the CGM) for extracting information/numbers from those reports.

## Author Contributions

All authors made substantial contributions to manuscript conception. Paula Jorge, Rosário Santos, Maria Luz Silva designed the study, Maria Manuela Mota-Freitas collected and gathered data, Paula Jorge and Maria Manuela Mota-Freitas wrote the paper, Gabriela Soares and Ana Maria Fortuna provided clinical data, and together with Maria Luz Silva and Rosário Santos provided conceptual advice and critical revisions. All authors analyzed and discussed the results as well as interpretations at all stages.

## Conflicts of Interest

The authors declare no conflict of interest.
